# Identification of three single nucleotide polymorphisms in *Anopheles gambiae* immune signaling genes that are associated with natural *Plasmodium falciparum* infection

**DOI:** 10.1186/1475-2875-9-160

**Published:** 2010-06-11

**Authors:** Ashley A Horton, Yoosook Lee, Cheick A Coulibaly, Vanessa K Rashbrook, Anthony J Cornel, Gregory C Lanzaro, Shirley Luckhart

**Affiliations:** 1 Department of Medical Microbiology and Immunology, School of Medicine, University of California, Davis, CA 95616, USA; 2 Department of Pathology, Microbiology and Immunology, School of Veterinary Medicine, University of California, Davis, CA 95616, USA; 3 Malaria Research and Training Center, University of Bamako, Mali; 4 DNA Technologies Core Facility, Genome Center, University of California, Davis, CA 95616, USA; 5 Mosquito Control Research Laboratory, Department of Entomology, University of California, Davis, 9240 S Riverbend Avenue, Parlier CA 93648, USA

## Abstract

**Background:**

Laboratory studies have demonstrated that a variety of immune signaling pathways regulate malaria parasite infection in *Anopheles gambiae*, the primary vector species in Africa.

**Methods:**

To begin to understand the importance of these associations under natural conditions, an association mapping approach was adopted to determine whether single nucleotide polymorphisms (SNPs) in selected immune signaling genes in *A. gambiae *collected in Mali were associated with the phenotype of *Plasmodium falciparum *infection.

**Results:**

Three SNPs were identified in field-collected mosquitoes that were associated with parasite infection in molecular form-dependent patterns: two were detected in the *Toll5B *gene and one was detected in the gene encoding insulin-like peptide 3 precursor. In addition, one infection-associated *Toll5B *SNP was in linkage disequilibrium with a SNP in sequence encoding a mitogen-activated protein kinase that has been associated with Toll signaling in mammalian cells. Both *Toll5B *SNPs showed divergence from Hardy-Weinberg equilibrium, suggesting that selection pressure(s) are acting on these loci.

**Conclusions:**

Seven of these eight infection-associated and linked SNPs alter codon frequency or introduce non-synonymous changes that would be predicted to alter protein structure and, hence, function, suggesting that these SNPs could alter immune signaling and responsiveness to parasite infection.

## Background

The causative agents of malaria are protozoan parasites of the genus *Plasmodium*, which are transmitted to humans by anopheline mosquitoes. The highest number of cases occurs in sub-Saharan Africa, where the most deadly parasite *Plasmodium falciparum *is transmitted primarily by *Anopheles gambiae sensu stricto*. With over 500 million new cases a year [1] and over half of the world's population at risk for malaria, increased understanding of the complex factors governing transmission is a pressing need.

Extensive genetic structuring among natural populations of *A. gambiae *is likely to have an impact on parasite transmission. In particular, frequencies of paracentric inversions on the right arm of the second chromosome (2R) have revealed that many *A. gambiae *populations deviate strongly from Hardy-Weinberg Equilibrium. On the basis of these findings, Mopti, Savanna, and Bamako chromosomal forms were described [2]. Genetic differentiation among these chromosomal forms revealed the presence of molecular forms characterized by fixed nucleotide differences in the intergenic spacer of the X-linked ribosomal DNA [3]. In Mali, the M molecular form corresponds to the Mopti chromosomal form and the S molecular form corresponds to the Savanna and Bamako forms. The distribution of the M molecular form appears mostly limited to West and Central Africa, whereas the S molecular form is found throughout the range of *A. gambiae *[4]. Gene flow between the S and Mopti-M forms is severely restricted [5,6].

In addition to chromosomal complexity, the genome of *A. gambiae *is characterized by high level nucleotide polymorphism. The published genome of *A. gambiae *PEST strain was reported to have a frequency of single nucleotide polymorphisms (SNPs) of 1.6 × 10^-3 ^or approximately 1 SNP per 625 bp [7]. Morlais *et al *[8] found similarly high levels of polymorphism in other laboratory strains of *A. gambiae*. The most recent genome assembly of *A. gambiae *in the *Ensembl *database [9] reports a SNP density of 1 per 247 bp.

In 35 genes putatively involved in mosquito-pathogen interactions and behaviour, Morlais *et al *[8] identified 460 SNPs, 140 of which encode nonsynonymous substitutions. The authors also examined SNP frequencies in genes encoding fibronectin, thioester-containing protein 3 (TEP3), and peptidoglycan recognition protein (PGRP) in field-caught *A. gambiae *from sites in Senegal, Burkina Faso and Cameroon where the M and S molecular forms are sympatric [8]. Although the genes selected by Morlais *et al *[8] have been shown to function in anti-pathogen defense in *A. gambiae *[10], no data on SNP associations with parasite infection and molecular form - a key variable based on aforementioned predictions of gene flow [5,6]- were presented.

In addition to studies that have reported SNPs in genes implicated in anti-parasite immunity, a number of studies have taken a genome-wide approach to identify factors that regulate parasite transmission. In particular, Riehle *et al *[11] determined that colocalized quantitative trait loci (QTL) within a region of chromosome 2L in *A. gambiae *collected from a single site in Mali accounted for a significant amount of the variation in *P. falciparum *infection. The authors designated these QTL as a *Plasmodium*-resistance island or PRI, but also acknowledged that there were numerous other loci throughout the genome that likely contributed to variation in infection [11]. Riehle *et al *[11] also hypothesized that resistance is the ancestral phenotype, whereas susceptibility to infection with *P. falciparum *results from mutations in the *A. gambiae *genome that result in failure of anti-parasite immunity.

Based on these general concepts - that *A. gambiae *populations exhibit significant genetic structuring, that SNPs are present in anti-pathogen genes in natural populations of *A. gambiae*, that susceptibility to infection may result from a failure of anti-parasite immunity- and the use of association mapping to link SNPs in functional genes with disease states [12], it was hypothesized that SNPs predicted to alter immune signaling could be linked to natural *P. falciparum *infection in *A. gambiae*. Furthermore, the strength of these associations may be a reflection of the different genetic backgrounds of *A. gambiae*, a phenomenon that will dictate the success of genetic interventions (e.g., creation of refractory mosquito strains) to block parasite transmission in natural populations [13].

To begin testing these hypotheses, SNPs in a subset of immune signaling genes were identified using direct sequencing of conserved domains, and the association of these SNPs with *P. falciparum *infection in *A. gambiae *collected in Mali were analysed. This association study revealed significant molecular form-dependent associations of *P. falciparum *infection with three SNPs in genes encoding Toll5B and an insulin-like peptide 3 precursor. In addition, these analyses revealed an epistatically associated SNP in a gene encoding MKK4 - a mitogen-activated protein kinase that has been predicted to function in the Toll signaling pathway [14-16]. Hence, these data support the proposed hypotheses and provide new insights on regulatory factors that are associated with control of *P. falciparum *infection in *A. gambiae*.

## Methods

### Sample collection

Blood fed female mosquitoes were collected from five villages in Mali in 2006 and 2007 (Table 1). These villages were selected based on previous data, which identified them as populations in which M and S forms are sympatric. This facilitated acquisition of adequate numbers of each form from a single environment. Mosquitoes were collected between July 28 and September 2 in 2006 and between August 8 and September 8 in 2007 to reduce genotype frequency changes related to season. Collected mosquitoes were dissected so that the head and thorax were separated from the abdomen. Head and thorax samples were subjected to enzyme-linked immunosorbent assay (ELISA) to determine *P. falciparum *infection status and the paired abdomens were used for species identification and SNP genotyping.

**Table 1:** Sample collection information by village (N = field-collected *A. gambiae*).

	Latitude	Longitude	**N**_**infected**_	**N**_**total**_	Infection rate *(2 year average)*
Bancoumana	12.200000	-8.266667	8	170	4.71%

Doneguebougou	12.80683	-7.98476	24	159	15.09%

Selinkenyi	11.70000	-8.28333	19	581	3.27%

Sidaribougou	11.466427	-5.743043	42	330	12.72%

Pimperena	11.46667	-5.70000	41	721	5.69%

### Species identification and ELISA for *P. falciparum *infection

Mosquitoes were identified to species using the PCR assay described by Scott *et al *[17] and to molecular form using the restriction fragment length polymorphism protocol of Fanello *et al *[3]. Briefly, following HhaI restriction of an amplified fragment of the X-linked ribosomal DNA, M molecular form individuals were identified by the presence of a 367 bp fragment, while S molecular form individuals were identified by fragments of 110 bp and 257 bp [3]. The presence of *P. falciparum *in heads and thoraces was determined using a "sandwich" circumsporozoite protein (CSP)-based ELISA [18,19]. All ELISA-positive specimens were rescreened against a standard curve of recombinant CSP to estimate relative infection levels.

### SNP discovery

Genes encoding Toll5B, MKK4 and insulin-like peptide precursor 3 were selected for analysis based on established roles in anti-pathogen signaling in *A. gambiae *or in other mosquito species [20-25]. Primers were designed to amplify fragments of the encoding sequences of the genes of interest in the size range of 120-260 bp, which was optimal for Luminex genotyping (see below).

For PCR, genomic DNA was isolated from *A. gambiae *abdominal tissue using the Qiagen BioSprint 96 (Valencia, CA) following the manufacturer's protocol. Each amplification reaction contained 1× buffer with MgCl_2 _(Roche), 67 μM of each dNTP (Applied Biosystems, Foster City, CA), 0.22 μM primers (Sigma-Aldrich, St. Louis, MO), 0.7 U Taq Polymerase (Roche), and 40 ng of genomic DNA. Cycling conditions were as follows: 94°C for 2 min; 2 cycles of 94°C for 30 sec, 62°C for 30 sec, 72°C for 30 sec; 6 cycles of 94°C for 30 sec, 55-61°C (the range of target-specific annealing temperatures) for 30 sec, 72°C for 30 sec; 27 cycles of 94°C for 30 sec, 55°C for 30 sec, 72°C for 30 sec, followed by a 7 minute extension at 72°C. Amplimer sizes were confirmed by electrophoresis prior to purification with Exo SapIT ([26]; USB, Cleveland, OH) for DNA sequencing.

Initial amplifications were performed using six sets of pooled genomic DNA samples from each of four *P. falciparum*-infected *A. gambiae *(n = 24) and six sets of pooled genomic DNA samples from each of four uninfected control *A. gambiae *(n = 24) collected in the same village on the same day. This pooling strategy has been shown to be very accurate (>98%) for detecting SNPs in individuals [27]. Pooled sequences were aligned using MegAlign (DNAStar Lasergene 8.0, Madison, WI) software and the ClustalW algorithm [28]. Each potential SNP was confirmed manually with the chromatogram of the pool. When a polymorphism was detected in the pooled *A. gambiae *amplimers, each of the four genomic DNAs in the pool was subjected to separate re-amplification and sequencing to determine if the locus was indeed polymorphic, and if so, which individuals were polymorphic and whether the individuals were homozygous or heterozygous.

Individual sequences from the 48 genomic DNA samples used for SNP discovery were aligned using MegAlign (DNAStar Lasergene 8.0, Madison, WI) software and the ClustalW algorithm [28]. Loci demonstrating divergence from the consensus sequences were designated as potential SNPs. The existence of each SNP was confirmed by manual examination of individual chromatograms. All SNPs were analysed by SIFT [29] and pMUT [30] to determine the likelihood that a particular SNP would affect protein function.

### Genotyping

SNPs for Luminex analysis [31] were chosen based on the presence of a minimum of 20 bp of flanking sequence that was free of SNPs. Based on this requirement, we selected 11 SNP loci out of 96 SNPs for analysis: insulin-like peptide 3 precursor loci 2-5 (AGAP010602; Ins32, Ins33, Ins34, Ins35); MKK4 loci 1 and 3 (AGAP003365; MKK41, MKK43) and Toll5B loci 1-4 and 6 (AGAP010669; Toll5B1, Toll5B2, Toll5B3, Toll5B4, Toll5B6). Allele-specific primers were designed for each of the 11 SNPs (Additional file 1). A total of 134 *P. falciparum*-infected *A. gambiae *and 134 uninfected *A. gambiae *collected in the same village on the same day were analysed with Luminex (Additional file 1). The 48 individuals used for SNP discovery were included among these samples, providing internal quality control checks for Luminex performance in detecting heterozygotes and homozygotes at each of the dimorphic loci.

### Allele-specific primer extension (ASPE)

For each Luminex assay, SNP-containing amplimers were combined in a final volume of 10 *μ*l. This mixture was treated with shrimp alkaline phosphatase (SAP) and exonuclease (*Exo*I) to remove unused PCR primers and dNTPs. For each reaction, 1 U of SAP (Amersham Biosciences, Inc., Piscataway, New Jersey) and 5 U *Exo*I (USB Corp., Cleveland, Ohio) were mixed with 10 *μ*l of amplified product and incubated at 37°C for 30 minutes, followed by 80°C for 15 minutes to inactivate the enzymes.

Ten *μ*l aliquots of ASPE master mix containing 500 nM of each ASPE primer, 50 mM MgCl_2_, 10× buffer (supplied with enzyme), 100 *μ*M each dATP, dCTP and dGTP, 400 *μ*M biotin-dCTP (Invitrogen, Carlsbad, California) and 0.8 U Platinum *Tsp *DNA Polymerase (Invitrogen) were dispensed to the pooled, SAP/*Exo*I-treated PCR products using a Biomek^® ^2000 Laboratory Automation Workstation (Beckman Coulter, Fullerton, California). The ASPE reaction was performed in a PTC-225 Peltier Thermal Cycler (MJ Research, Watertown, Massachusetts) and cycling parameters consisted of an initial denaturation at 96°C for 2 minutes, followed by 94°C for 30 seconds and 56°C for 2 minutes, repeated 49 times, ending with 72°C for 5 minutes.

### Hybridization to FlexMAP beads

Individual FlexMAP™ bead types (MiraiBio, Alameda, California) were obtained at a concentration of 2.5 × 10^5 ^microspheres/ml. After resuspension by vortexing for 5 minutes, 2.5 *μ*l of each bead type per sample was used to make up the appropriate bead mix for each of the three multiplex assays. Each bead mix was concentrated by centrifugation at 10,000 × *g *for three minutes followed by careful removal of the supernatant. Beads were then resuspended in 2× TM hybridization buffer (0.4 M NaCl, 0.2 M Tris HCl pH 8.0, 0.16% Triton X-100) and dH_2_O, and added to each sample for a final volume of 50 *μ*l (resulting in 625 beads/allele in 1× TM). Samples were denatured at 96°C for 90 seconds, followed by hybridization at 52°C, 47°C and 37°C for 30 minutes at each temperature. The hybridized beads were washed twice by centrifugation at 3,000 × *g *for 3 minutes, removal of the supernatant and resuspension in 70 *μ*l of 1× TM buffer. After centrifugation and removal of the supernatant for a third time, beads were resuspended in 70 *μ*l 1× TM buffer containing 8 *μ*g/ml streptavidin-phycoerythrin (ProZyme, San Leandro, California), a fluorescent reporter molecule used to detect the ASPE-incorporated biotin.

### Sample analysis on the Luminex 100 System

Samples were analysed on the Luminex 100 System using Data Collection Software Version 1.7 with settings specified by the manufacturer; the median fluorescence intensity (signal) was measured over 100 independent events (beads). The genotype of each SNP locus was determined by the ratio of fluorescence intensity of allele A, *I_A_*, and that of allele B, *I_B_*. If *I_A _/I_B _*was greater than 3.5, genotype was set as AA; if *I_A _/I_B _*was less than 0.5, genotype was set as BB; for other ratio values, the genotype was set as AB. These thresholds were confirmed by comparison to the direct sequencing data available for each of the polymorphic loci.

### Statistics

The *pwr *package for R http://cran.r-project.org/web/packages/pwr/index.html was used for power analysis. This package calculated effect size, required sample size, and power for each SNP using the methods of Cohen [32]. Significant differences in allele frequency (1) between infected and uninfected samples within a population and (2) between any two populations were determined using Fisher's exact test (for 2 by 2 contingency tables) or Chi-square test implemented in R statistics package http://www.r-project.org. Significance thresholds were adjusted for multiple comparisons [33].

Unaccounted population structure can lead to the discovery of spurious associations or dilute true associations [34,35]. To eliminate associations due to population structure, we tested whether our SNP genotypes showed significant divergence based on geographic location and molecular form. Samples were first divided into eight groups based on collection site and molecular form. Next, pairwise F_ST _values were calculated using Arlequin v 3.11 [36]. The two groups with the highest p-values were then combined and pair-wise F_ST _values were recalculated. If genetic divergence, as described by F_ST_, between a pair of groups is minimal, the p-value is close to 1. This was repeated until all between-group F_ST _values were significant. The eight initial groups (based on collection site and molecular form) were ultimately reduced to 3 distinct groups: M, S1, and S2, as described in the Results. Within each of the three groups, linkage disequilibrium was calculated using Arlequin. Phylip v 3.68 [37,38] was used for phylogenetic tree construction.

## Results

Among the 11 SNPs analysed, the frequency of exact matches between direct sequencing and Luminex for individual genotypes (n = 48) was greater than 98%, indicating that Luminex was highly accurate for genotyping.

**Figure 1:**
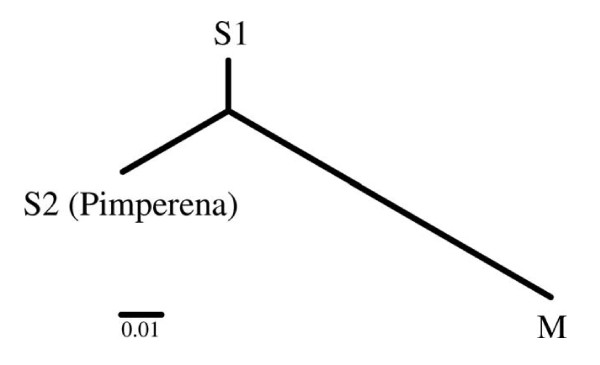
**Phylogenetic tree of field-collected *A. gambiae***. Construction was based on pairwise F_ST _calculated from SNP allele frequencies.

Based on SNP genotypes at the 11 loci selected, at least three genetically distinct populations in Mali (Figure 1) were identified. In agreement with population genetic studies based on microsatellites [39,40], significant genetic differentiation between M and S molecular forms was observed. Further, Pimperena S (S2) form mosquitoes were differentiated from S forms collected in other villages (S1). This outcome may be due to temporal variation in the relative abundance of the Bamako and Savanna chromosomal forms, both of which are the S molecular form and therefore indistinguishable using the Fanello *et al *[3] molecular form diagnostic.

The association between SNP genotypes at each locus and infection status within each of the three populations indicated in Figure 1 was evaluated using Chi-square and Fisher's exact tests. Infection rates of the genotyped *A. gambiae *ranged from 3.27% in Selinkenyi to a high of 15.09% in Doneguebougou (Table 1). Genotype frequencies at the Ins35 locus were significantly different between the Pimperena (S2) and S1 groups, while genotype frequencies at the Toll5B1 locus were significantly different between S2 and all other groups (M and S1 in Table 2). All SNPs identified to be associated with infection status had an effect size (h) greater than 1 (Table 3) and power greater than 0.85. Those SNPs that were determined to be associated with population groups had power greater than 0.83 (Table 3). SNPs with smaller h, such as Ins32 and Ins35 (0.75 < h < 1), could be associated with infection if a larger sample size was scored. For example, if 30 infected specimens and 62 uninfected specimens for Ins32 were genotyped and a similar genotype frequency distribution was observed, Ins32 could be identified as significantly associated with infection status. The sample sizes of this study were not sufficient to confirm involvement of SNPs with smaller h - such as Ins32 or Ins35 - in the regulation of *P. falciparum *development in *A. gambiae*.

**Table 2:** Genotype abundance for uninfected and *P. falciparum*-infected molecular form *A. gambiae *associated with *P. falciparum *infection status.

Chromosome	Gene Location	Locus	Genotype	Mutation Type	^a^M		^b^S1		^c^S2	
					
					^d^UI	^e^I	UI	I	UI	I
3L	7,103,451-7,105,904	Ins34*	**CC**	synonymous	**17**	**6**	20	**19**	25	18
			
			**CT**	synonymous	**8**	**19**	23	33	10	18
			
			**TT**	synonymous	**5**	**0**	7	6	3	1

3L	8,174,795-8,178,377	Toll5B1*^1^	**CC**	synonymous	**1**	**18**	**6**	**47**	29	35
			
			**TC**	synonymous	**29**	**7**	**43**	**11**	9	2
			
			**TT**	synonymous	**0**	**0**	**1**	**0**	0	0

3L	8,174,795-8,178,377	Toll5B6*	**AA**	nonsynonymous	2	4	**4**	**4**	0	5
			
			**AG**	nonsynonymous	5	2	18	3	13	12
			
			GG	nonsynonymous	23	19	28	51	25	20

3L	7,103,451-7,105,904	Ins35**^1^**	CC	nonsynonymous	27	17	40	45	37	35
			
			CT	nonsynonymous	3	8	10	13	1	1
			
			TT	nonsynonymous	0	0	0	0	0	1

3L	7,103,451-7,105,904	Ins32	AA	synonymous	2	0	5	1	2	1
			
			AG	synonymous	9	16	25	29	11	17
			
			GG	synonymous	19	9	20	28	25	19

3L	7,103,451-7,105,904	Ins33	CC	nonsynonymous	24	14	29	30	1	1
			
			CT	nonsynonymous	5	11	20	25	10	9
			
			TT	nonsynonymous	1	0	1	3	27	27

2R	36,863,007-36,864,732	Mkk43	AA	synonymous	0	0	1	0	0	0
			
			AG	synonymous	5	7	10	3	1	4
			
			GG	synonymous	25	18	39	55	37	33

3L	8,174,795-8,178,377	Toll5B2	AA	nonsynonymous	30	25	46	56	37	36
			
			AC	nonsynonymous	0	0	4	2	1	1
			
			CC	nonsynonymous	0	0	0	0	0	0

Groups included M uninfected, M infected, S1 uninfected, S1 infected, S2 uninfected, S2 infected. ^a^M forms collected in Doneguebougou, Selinkenyi, and Sidaribougou, ^b^S forms collected in Bancomana, Doneguebougou, Selinkenyi and Sidaribougou, ^c^S forms collected in Pimperena. ^d^Uninfected (UI); ^e^Infected (I) with *P. falciparum*.

*Significant association with infection status (p < 0.0014), numbers of individuals in **bold**. ^1^Significant difference in genotype frequencies between S1 and S2 (p < 0.0014).

**Table 3:** Summary of power analysis.

	M: infected (inf) vs uninfected (uninf)				S1: infected vs uninfected				S2: infected vs uninfected			
	**h^a^**	**N_inf _^b^**	**N_uninf _^b^**	**power^c^**	**h^a^**	**N_inf _^b^**	**N_uninf _^b^**	**power**	**h^a^**	**N_inf _^b^**	**N_uninf _^b^**	**power^c^**

Ins32	0.898	30	62	0.548	0.443				0.434			

Ins33	0.615	65	127		0.440				0.317			

**Ins34**	**1.146**	**21**	**30**	**0.85**	0.254				0.563	77	154	

Ins35	0.780	40	81		0.481				0.288			

Mkk41	0.694	51	100		0.452				0.634	61	120	

Mkk43	0.340				0.583	72	143		0.636	60	122	

**Toll5B1**	**1.558**	**10**	**20**	**0.995**	**2.116**	**6**	**9**	**1**	0.344			

Toll5B2	-				0.269				0.344			

Toll5B3	0.576	74	145		0.357				0.485			

Toll5B4	0.380				0.415				0.321			

Toll5B6	0.514	83	183		1.292	**15**	**27**	1	0.627	62	124	

Actual number genotyped		25	30			58	50			37	38	

	M vs S1				S1 vs S2				M vs S2			

	h^a^	M^b^	S1^b^	power^c^	h^a^	S1^b^	S2^b^	power	h^a^	S1^b^	S2^b^	power^c^

Ins32	0.085				0.305				0.289			

Ins33	0.348				0.415				0.192			

Ins34	0.155				0.465				0.423			

**Ins35**	0.270				**0.626**	**93**	**75**	**0.834**	0.701	**55**	83	0.775

**Mkk41**	**0.755**	**55**	**59**	**0.914**	0.241				**0.745**	**55**	**62**	**0.842**

Mkk43	0.354				0.261				0.566	90	116	0.497

**Toll5B1**	**0.344**				**0.737**	**75**	**50**	**0.956**	**0.870**	**55**	**35**	**0.956**

Toll5B2	0.151				0.091				-			

Toll5B3	0.178				0.294				0.347			

Toll5B4	0.550	81	160	0.55	-				0.561	90	121	0.486

Toll5B6	-				0.384				0.456			

Actual number genotyped		55	108			108	75			55	75	

^a^Effect size. ^b ^For SNPs for which effect size was greater than 0.5, required sample size to achieve 80% power was calculated. ^c ^Power given the actual number of genotyped samples. If the actual number of genotyped samples exceeded the required sample size, SNPs are marked in underlined bold text.

Three SNPs - Ins34 (3L), Toll5B1 (3L), and Toll5B6 (3L) (Table 2) - were found to be associated with *P. falciparum *infection status. Ins34 is a synonymous SNP resulting in a change from GGC to GGT at nucleotide position 462 in the *Insulin-like peptide 3 precursor *gene. Toll5B1 introduces a synonymous SNP at nucleotide position 129, changing the codon from ATC, a common codon (28.3%), to ATT, a rarer codon (12.9%). Toll5B6 results in a non-synonymous codon change from AGC (Ser, S) to AAC (Asn, N) at amino acid position 454 in the predicted translation of *Toll5B*. These SNPs were not identified in either the NCBI or Anobase SNP databases generated by reads from the PEST strain (10× coverage) or from the MOPTI strain (1.2× coverage) aligned to Celera *A. gambiae *contigs, although Ins34 and Toll5B6 were both present in the M and S form scans available from VectorBase [41]. However, these loci were not identified to be polymorphic in either scan [41]. The CC genotype at the Ins34 locus in M form mosquitoes was more common in samples that were not infected with *P. falciparum*. In both M and S1 populations, Toll5B1 C alleles were significantly associated with infection status. While the Toll5B1 TT genotype was rare, TC heterozygotes were more common in uninfected *A. gambiae *than in *P. falciparum*-infected *A. gambiae*. Within the S1 population, individuals with the AG genotype at the Toll5B6 locus were less likely to be infected with *P. falciparum*.

**Figure 2:**
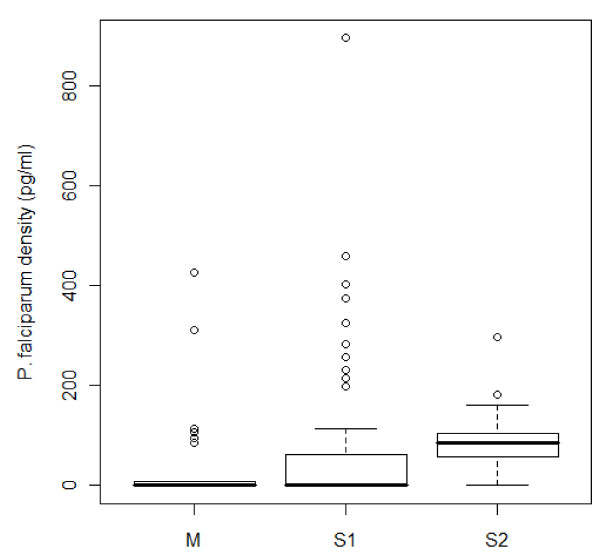
***P. falciparum *density (pg/ml) for field-collected M, S1 and S2 molecular form *A. gambiae***.

Significant differences in median *P. falciparum *sporozoite infection intensities were observed in the collected samples (Kruskal-Wallis rank sum test, p = 1.88x10^-5^, Figure 2, Table 4). However, no significant association between *P. falciparum *infection intensity and any SNP locus genotype was observed. The sporozoite infection intensities were highly variable in M and S1 populations as indicated by the standard deviations being more than double their associated means shown in Table 4. Among infected mosquitoes, samples from Pimperena (S2) had higher median sporozoite intensities than those from other villages. VectorBase *A. gambiae *population data indicated that samples from Pimperena are mostly Savanna chromosomal form while other sites like Doneguebougou and Selinkenyi are composed of a mixture of Bamako and Savanna forms. Thus, the possibility of chromosomal form affecting the density of *P. falciparum *in the S1 group cannot be ruled out.

**Table 4:** Relative *P. falciparum *(Pf) sporozoite densities in infected *A. gambiae *as determined by Pf CSP ELISA.

Population	N_infected_	Pf density median	**Pf density mean ± s.d**.	Bamako/Savanna ratio*
**M**	25	0.4405	41.69186 ± 100.23573	

**S1**	58	0.5865	71.23893 ± 156.89776	1.7292

**S2**	37	85.5000	84.23959 ± 58.04953	0.0137

*Estimated from *A. gambiae *data in VectorBase Population Data page.

## Discussion

Many factors contribute to mosquito infection and successful transmission of malaria parasites, including innate immunity. As such, considerable efforts have been focused on understanding the mosquito immune system and natural variability in these defenses. In humans, numerous reports have demonstrated that natural variability to infection can be associated with single nucleotide polymorphisms (SNPs) in genes that regulate host immunity [42-44]. Although SNP associations have been described for a variety of human infections and diseases, these have not been identified for *P. falciparum *infection in genetically defined natural populations of *A. gambiae *until this study.

The Toll and Imd signaling pathways are important regulators of innate immunity in *A. gambiae*. In particular, Garver *et al *[45] reported that, under laboratory conditions, the Toll pathway controlled *P. falciparum *infection intensity in *A. gambiae*, while the Imd pathway appeared to regulate resistance to infection. Two *Toll5B *SNPs - Toll5B1 and Toll5B6 - were significantly associated with *P. falciparum *infection status among field-collected *A. gambiae*. *Anopheles gambiae Toll5B *is orthologous to *Drosophila melanogaster Toll-5*, also known as *Tehao *[10], and *Toll5 *in *Aedes aegypti *[46]. In *Ae. aegypti*, *Toll5B *was inducibly expressed in the mosquito fat body following fungal infection [47]. In *A. gambiae*, Pinto *et al *[48] reported that *Toll5B *expression is significantly upregulated in hemocytes of adult female mosquitoes infected with *Plasmodium berghei *at 24-28 hours post-infection, a period associated with active parasite invasion of the midgut epithelium. Based on these observations and these data, *Toll5B *is likely to be responsive to *P. falciparum *infection in *A. gambiae *under natural conditions.

Both *Toll5B *SNPs showed divergence from Hardy-Weinberg equilibrium. In particular, Toll5B1 showed significant divergence from Hardy-Weinberg expectation in both the M (p = 0.00016) and S1 (p = 0.00077) forms, while Toll5B6 showed significant divergence from Hardy-Weinberg expectation in the M form (p = 0.00035; Table 5), suggesting that selection pressure(s) acting on these loci may skew the genotype frequencies of this gene. For Toll5B1, the observed heterozygosity was greater than expected, both in M and S1 groups (Table 5). For this SNP, CT heterozygotes were more common (96%) in uninfected samples while 72% of infected samples were CC homozygotes. TT homozygotes were very rare and only one sample of this genotype was identified in the entire collection. These circumstances indicate the possibility of heterosis or balancing selection on this locus. For Toll5B6, the GG homozygote was the most common form in all groups and the proportion of GG was highest in the M uninfected group. The effect size of this SNP was 0.514, so a larger sample size (83 infected and 183 uninfected) would be needed (Table 3) to confirm involvement of this SNP with respect to infection status in the M form population.

**Table 5:** Hardy-Weinberg Exact Test result (p-values).

	M			S1			S2		
**HWE**	**Hobs**	**Hexp**	**pvalue**	**Hobs**	**Hexp**	**pvalue**	**Hobs**	**Hexp**	**pvalue**

Ins32	0.43636	0.40033	0.73394	0.50926	0.42993	0.07098	0.37333	0.36725	1

Ins33	0.23636	0.31176	0.0868	0.37037	0.4112	0.34935	0.24	0.2885	0.21444

Ins34	0.49091	0.45054	0.55952	0.51852	0.47321	0.41286	0.37333	0.36725	1

Ins35	0.2	0.18165	1	0.18519	0.16882	0.59682	0.02667	0.05226	0.0395

Mkk41	0.43636	0.4844	0.57431	0.43342	0.51337	0.51337	0.33333	0.42282	0.09584

Mkk43	0.21818	0.19616	1	0.12037	0.12984	0.41148	0.06667	0.06488	1

Toll5B1*	***0.65455***	***0.44437***	**0.00016**	***0.5***	***0.38587***	**0.00077**	0.14667	0.13682	1

Toll5B2	0.41818	0.43786	1	0.05556	0.05426	1	0.02667	0.02648	1

Toll5B3	0.49091	0.47189	0.76152	0.33333	0.43342	0.02355	0.41333	0.41172	1

Toll5B4	0.49091	0.47189	1	0.37963	0.48678	0.02534	0.42667	0.48322	0.34206

Toll5B6*	***0.12727***	***0.28841***	**0.00035**	0.19444	0.28523	0.00189	0.33333	0.36018	0.52328

P-values below the significance threshold of 0.0014, adjusting for multiple comparison, are highlighted in bold text. * Locus departed from H-W expectation.

Toll5B1 occurs in a highly polymorphic region, containing 13 polymorphic loci within a stretch of 166 bp. Six of these SNPs occur in clusters of rare codons or result in the change from a common codon to a rare one. Clusters of rare codons have been shown to alter protein production in a synergistic manner [49-51]. Genotype frequencies of Toll5B1 and infection associations of this SNP were significantly different between Pimperena (S2) and *A. gambiae *M and S1 groups (Table 2). Taken together with observations on codon frequency and protein function, these data suggest that within the M and S1 molecular forms, this SNP may have a functional effect on *P. falciparum *infection, but this association may be population-specific, as indicated by the lack of any Toll5B1-*P. falciparum *infection association in the Pimperena (S2) population. Toll5B6 is a non-synonymous SNP that introduces the amino acid change S454N. The replacement of serine by the more bulky asparagine could introduce subtle changes in the 3-dimensional structure of the Toll leucine-rich repeat (LRR) in which this mutation occurs.

Additional analyses revealed that MKK43, a SNP in the MAPK kinase *MKK4 *gene, located on chromosome 2, was in linkage disequilibrium with infection-associated Toll5B1, a SNP on chromosome 3, in the S1 population (p = 0.0001). In mammals, MKK4 can be activated by TLR3 [16] and TLR2 signaling [14,15], indicating that MKK4 activation is functionally linked to Toll signaling. Downstream of this activation, MKK4 functions with MKK7 as the primary activator of c-Jun N-terminal kinase or JNK, one of three immunity-associated MAPKs. In *D. melanogaster*, Toll activation of MKK4/7 and JNK during septic injury regulates cytoskeletal genes typically associated with a wound healing response to infection [52]. A potential Toll/MKK4/JNK signaling module - if biologically functional in *A. gambiae *- could be linked to the profound cytoskeletal changes in the midgut epithelium that have been described during *P. berghei *and *P. falciparum *infection of laboratory and field specimens of *A. gambiae*, respectively [53-55]. This possibility is currently being investigated.

In mammals, the insulin/insulin-like growth factor signaling cascade (IIS) has been shown to regulate innate immunity through Toll- and NF-κB-dependent pathways [56,57]. In particular, IIS activation can induce or inhibit NF-κB-dependent signaling and is, thereby, capable of exerting both pro- and anti-inflammatory effects on the host immune response. In *Anopheles stephensi*, control of malaria parasite development is regulated by signaling proteins associated with the IIS cascade [[22], Corby-Harris *et al*. unpublished]. The IIS is highly conserved [23] and critical components of the cascade, as well as a variety of insulin-like peptides, including insulin-like peptide 3 precursor, are expressed in the midgut of *A. gambiae *[[58]; Luckhart, unpublished].

Four SNPs in the *insulin-like peptide 3 precursor *gene - Ins32, Ins33, Ins34 and Ins35 - were analysed and significant infection (Ins34) and molecular form (Ins35) associations were found for two of these (Table 2). Ins34 introduces a synonymous mutation, with no remarkable change in codon frequency. Although Ins34 is predicted to have little to no effect on protein function, this infection-associated SNP was in linkage disequilibrium in the S1 population with Toll5B2 (p = 0.03307, Additional file 2), a SNP that introduces the non-synonymous mutation D56A into Toll5B. This SNP encodes a mutation close to the N-terminus and outside of the predicted LRRs, in an ectodomain region identified in other Toll proteins as the cysteine-rich capping structure [59]. The N-terminal capping structure may participate in protein-protein interactions that are critical for Toll receptor function [59].

Within the S1 population, Ins32, Ins33, and Ins35 showed levels of linkage disequilibrium with Toll5B2 similar to that observed for Ins34 (p = 0.01238, 0.02931, 0.00851, respectively, Additional file 2), which likely reflects the fact that these loci are closely linked physically. Although Ins35 was not significantly associated with infection status (Table 2), this SNP was also in linkage disequilibrium with Toll5B2 in the S2 population (p-value = 0.00406, Additional file 2). However, in the S2 population, the neighboring SNPs - Ins32, Ins33, Ins34 - did not show similar levels of linkage disequilibrium with Toll5B2 (p = 0.09584, 0.14396, 0.13238, respectively, Additional file 2), suggesting that the associations of Ins34 and Ins35 with Toll5B2 may, in fact, represent novel biological functionality that is population-specific in *A. gambiae*.

The positional effects of SNPs in the architecture of signaling cascades have been investigated in a series of relevant studies. Riley *et al *[60] examined SNPs in the *D. melanogaster *Ras-mediated signal transduction pathway. The least polymorphic signaling protein genes (e.g., *Ras, Dr*, and *Polehole*) were those that were proximal to the origin of signaling at the cell surface, while the most polymorphic genes were located farther downstream in the signaling cascade (e.g., *Dsor1, Csw *and *Ksr*; [60]). Computational simulations of MAPK signaling predicted similar constraints in that if signal amplification was crucial, the upstream signaling proteins were more constrained than were the downstream components [61]. Together these findings suggest that a SNP in an upstream component of a signaling cascade, such as Toll or an ILP, could have pleiotropic effects on the downstream components of the signaling cascade and an increased potential for a deleterious outcome [62]. In studies of the Tor signaling pathway, however, selection constraints were found to be greater in the downstream components rather than the upstream components of the cascade [62]. Regardless of the pattern of selection constraints, the polarity of a signal transduction pathway is an integral part of determining the effect of a SNP. As such, the molecular cell biology of the signaling pathway protein networks highlighted herein can complement studies of phenotype-associated SNPs in natural *A. gambiae *populations.

## Conclusions

In summary, the infection-associated SNPs that have been identified here could be used as genetic markers for the susceptibility of an anopheline population to malaria parasite infection. More importantly, however, these SNPs confirm previous laboratory associations of the target genes with the regulation of parasite infection in *A. gambiae*, extending possible functional linkages among these target genes and confirming the influence of population structuring on biological associations of the SNPs under study. In particular, these data together with associated laboratory data that implicate these gene products in anti-parasite immunity suggest that Toll5B, MKK4 and insulin-like peptide 3 alone or perhaps in some combination may be involved in the regulation of *P. falciparum *development in *A. gambiae *under natural conditions. The alternative hypothesis - that the true functional genes are linked to these SNPs - will be evaluated as haplotype maps are developed. In addition, these data have revealed that SNPs are not equally distributed among *A. gambiae *molecular forms and that selection pressures may be driving deviations from Hardy-Weinberg equilibrium. These insights suggest that gene flow may impede the spread of transgenes under field conditions and that selection may alter the distribution of transgenes, factors that must be accommodated in the development of any field strategies.

## Competing interests

The authors declare that they have no competing interests.

## Authors' contributions

AAH performed the SNP discovery, assisted with the Luminex genotyping, and prepared the manuscript; YL performed the statistical analyses and edited the manuscript; CAC performed the *P. falciparum *CSP ELISA and assisted with the SNP discovery; VKR performed the Luminex genotyping and edited the manuscript; AJC planned and directed the mosquito collections and edited the manuscript; GCL assisted with the design of the studies, planned the collections and edited the manuscript; SL assisted with the design of the studies and experimental plans, and helped draft the manuscript. All authors read and approved the final manuscript.

## Supplementary Material

Additional file 1 **Primer sequences used for direct sequencing and SNP genotyping**. Genomic primers: universal tag sequence (underlined) for DNA sequencing. Allele-specific Luminex primers: FlexMAP Bead TAG sequence (underlined) followed by 3' allele-specific sequence with terminal SNP nucleotide. Click here for file

Additional file 2 **Linkage disequilibrium information for all SNPs analysed**. Click here for file
