# Supplementary material for: Identification of three single nucleotide polymorphisms in Anopheles gambiae immune signaling genes that are associated with natural Plasmodium falciparum infection
Source: Malar J. 2010 Jun 11;9:160. doi: 10.1186/1475-2875-9-160 (PMC2896950; doi:10.1186/1475-2875-9-160)
Supplement: Additional file 1 — Primer sequences used for direct sequencing and SNP genotyping. Genomic primers: universal tag sequence (underlined) for DNA sequencing. Allele-specific Luminex primers: FlexMAP Bead TAG sequence (underlined) followed by 3' allele-specific sequence with terminal SNP nucleotide. [file 1475-2875-9-160-S1.DOC]

**Additional file 1. Primer sequences used for direct sequencing and SNP genotyping.**

| **Locus** | **Genomic primers (5’-3’)** | **Allele-specific Luminex primer sequence (5’-3’)** |
| --- | --- | --- |
| **Toll 5B1** | **F:GTAAAACGACGGCCAGTTGCCCAGGTTCAAATGTTC**  **R:CAGGAAACAGCTATGACCCGCTGATACCTCTGCATTG** | **T:TACACATCTTACAAACTAATTTCACAAATGTTCATCTCTCGATT**  **C:AATCATACCTTTCAATCTTTTACACAAATGTTCATCTCTCGATC** |
| **Toll5B2** | **F:GTAAAACGACGGCCAGTTGCCCAGGTTCAAATGTTC**  **R:CAGGAAACAGCTATGACCCGCTGATACCTCTGCATTG** | **C:AATCTAACAAACTCATCTAAATACCTTAATGTGACATGTTCCGC**  **A:CAATTAACTACATACAATACATACCTTAATGTGACATGTTCCGA** |
| **Toll5B3** | **F:GTAAAACGACGGCCAGTTGCCCAGGTTCAAATGTTC**  **R:CAGGAAACAGCTATGACCCGCTGATACCTCTGCATTG** | **G:TCAAAATCTCAAATACTCAAATCAGGGACCAACCCAATCTACGG**  **C:CTTTAATCCTTTATCACTTTATCAGGGACCAACCCAATCTACGC** |
| **Toll5B4** | **F:GTAAAACGACGGCCAGTTGCCCAGGTTCAAATGTTC**  **R:CAGGAAACAGCTATGACCCGCTGATACCTCTGCATTG** | **T:TCAATCAATTACTTACTCAAATACGATCTATCAAGACTGCCCAT**  **C:CTTTTACAATACTTCAATACAATCGATCTATCAAGACTGCCCAC** |
| **Toll5B6** | **F:GTAAAACGACGGCCAGTGGATGCGTTCACGTCTCAG**  **R:CAGGAAACAGCTATGACCCAATAGCATTAAACGAAAGATCCAG** | **G:TTACTCAAAATCTACACTTTTTCAACATCCGGGATTATTTTCAG**  **A:CTTTTCAATTACTTCAAATCTTCAACATCCGGGATTATTTTCAA** |
| **Mkk41** | **F:GTAAAACGACGGCCAGTCCGTACATGGCGGTAAGAGA**  **R:CAGGAAACAGCTATGACCGTAATTCCGAGCGACCACAC** | **C:** **TCAATCATCTTTATACTTCACAATAGAGAGAGAGGAGAGAGACC**  **T: TCATTTCAATCAATCATCAACAATAGAGAGAGAGGAGAGAGACT** |
| **Mkk43** | **F:GTAAAACGACGGCCAGTCCGTACATGGCGGTAAGAGA**  **R:CAGGAAACAGCTATGACCGTAATTCCGAGCGACCACAC** | **A:** **TATATACACTTCTCAATAACTAACCCGAACGCATCGATCCCCAA**  **G: CAATTTACTCATATACATCACTTTCCGAACGCATCGATCCCCAG** |
| **Ins32** | **F:GTAAAACGACGGCCAGTCCGATCTGCATACGACGAG**  **R:CAGGAAACAGCTATGACCACGATGCCACCACGATTC** | **A:CTTTTCATCTTTTCATCTTTCAATCCGCTGACAACTTCGCCAAA**  **G: TCAATCATTACACTTTTCAACAATCCGCTGACAACTTCGCCAAG** |
| **Ins33** | **F:GTAAAACGACGGCCAGTCCGATCTGCATACGACGAG**  **R:CAGGAAACAGCTATGACCACGATGCCACCACGATTC** | **C:CTATCTTCATATTTCACTATAAACACGGAGGACTGGATGAACGC**  **T: CTTTCAATTACAATACTCATTACAACGGAGGACTGGATGAACGT** |
| **Ins34** | **F:GTAAAACGACGGCCAGTCCGATCTGCATACGACGAG**  **R:CAGGAAACAGCTATGACCACGATGCCACCACGATTC** | **T:TCATTTACCAATCTTTCTTTATACAGCAGTCGCAGTCGGTCGGT**  **C: TCATTTCACAATTCAATTACTCAAAGCAGTCGCAGTCGGTCGGC** |
| **Ins35** | **F:GTAAAACGACGGCCAGTCCGATCTGCATACGACGAG**  **R:CAGGAAACAGCTATGACCACGATGCCACCACGATTC** | **T:TACATCAACAATTCATTCAATACATGGATGAACATGGTCTACCT**  **C: CTTCTCATTAACTTACTTCATAATTGGATGAACATGGTCTACCC** |

Genomic primers: universal tag sequence (underlined) for DNA sequencing. Allele-specific Luminex primers: FlexMAP Bead TAG sequence (underlined) followed by 3’ allele-specific sequence with terminal SNP nucleotide.
