# Supplementary material for: Identification of three single nucleotide polymorphisms in Anopheles gambiae immune signaling genes that are associated with natural Plasmodium falciparum infection
Source: Malar J. 2010 Jun 11;9:160. doi: 10.1186/1475-2875-9-160 (PMC2896950; doi:10.1186/1475-2875-9-160)
Supplement: Additional file 2 — Linkage disequilibrium information for all SNPs analysed. [file 1475-2875-9-160-S2.DOC]

**Additional file 2: Linkage disequilibrium information for all SNPs analysed.**

| **Significance Threshold** | **0.000932171** |  |  |  |  |  |  |  |  |  |  |
| --- | --- | --- | --- | --- | --- | --- | --- | --- | --- | --- | --- |
| **M** | **Ins32** | **Ins33** | **Ins34** | **Ins35** | **Mkk41** | **Mkk43** | **Toll5B1** | **Toll5B2** | **Toll5B3** | **Toll5B4** | **Toll5B6** |
| pvalue | 0 | 1 | 2 | 3 | 4 | 5 | 6 | 7 | 8 | 9 | 10 |
| 0 | - |  |  |  |  |  |  |  |  |  |  |
| 1 | 0.11752 | - |  |  |  |  |  |  |  |  |  |
| 2 | 0 | 0.07069 | - |  |  |  |  |  |  |  |  |
| 3 | 0.0001 | 0 | 0.00089 | - |  |  |  |  |  |  |  |
| 4 | 1 | 0.49426 | 0.22426 | 0.70673 | - |  |  |  |  |  |  |
| 5 | 0.2995 | 0.55307 | 0.68693 | 0.95416 | 0.36792 | - |  |  |  |  |  |
| 6 | 0.6004 | 0.99713 | 0.81446 | 0.92683 | 0.3803 | 0.20238 | - |  |  |  |  |
| 7 | 1 | 1 | 1 | 1 | 1 | 1 | 1 | - |  |  |  |
| 8 | 0.53921 | 0.47347 | 0.32495 | 0.68327 | 0.57713 | 0.8303 | 0.27881 | 1 | - |  |  |
| 9 | 0.55257 | 0.41396 | 0.44812 | 0.59485 | 0.14059 | 0.80762 | 0.51762 | 1 | 0 | - |  |
| 10 | 0.19178 | 0.35733 | 0.5299 | 0.53465 | 0.02941 | 1 | 0.79238 | 1 | 0.27802 | 0.65772 | - |
|  |  |  |  |  |  |  |  |  |  |  |  |
| **S1** | **Ins32** | **Ins33** | **Ins34** | **Ins35** | **Mkk41** | **Mkk43** | **Toll5B1** | **Toll5B2** | **Toll5B3** | **Toll5B4** | **Toll5B6** |
| pvalue | 0 | 1 | 2 | 3 | 4 | 5 | 6 | 7 | 8 | 9 | 10 |
| 0 | - |  |  |  |  |  |  |  |  |  |  |
| 1 | 0.82228 | - |  |  |  |  |  |  |  |  |  |
| 2 | 0 | 0.86515 | - |  |  |  |  |  |  |  |  |
| 3 | 0 | 0 | 0 | - |  |  |  |  |  |  |  |
| 4 | 0.48347 | 0.70455 | 0.12554 | 0.1705 | - |  |  |  |  |  |  |
| 5 | 0.05782 | 0.05386 | 0.23129 | 0.01881 | 0.9798 | - |  |  |  |  |  |
| 6 | 0.02228 | 0.84297 | 0.65842 | 0.40525 | 0.12198 | 0.0001 | - |  |  |  |  |
| 7 | 0.01238 | 0.02931 | 0.03307 | 0.00851 | 0.49832 | 0.00347 | 0.00604 | - |  |  |  |
| 8 | 0.73149 | 0.66921 | 0.98297 | 0.78614 | 0.22713 | 0.2505 | 0.83138 | 0.00772 | - |  |  |
| 9 | 0.54218 | 0.9897 | 0.49663 | 0.82347 | 0.76505 | 0.3401 | 0.25366 | 0.13921 | 0 | - |  |
| 10 | 0.88446 | 0.02703 | 0.55139 | 0.91356 | 0.57119 | 0.85337 | 0.06238 | 0.90505 | 0.79158 | 0.00832 | - |
|  |  |  |  |  |  |  |  |  |  |  |  |
| **S2** | **Ins32** | **Ins33** | **Ins34** | **Ins35** | **Mkk41** | **Mkk43** | **Toll5B1** | **Toll5B2** | **Toll5B3** | **Toll5B4** | **Toll5B6** |
| pvalue | 0 | 1 | 2 | 3 | 4 | 5 | 6 | 7 | 8 | 9 | 10 |
| 0 | - |  |  |  |  |  |  |  |  |  |  |
| 1 | 0.96762 | - |  |  |  |  |  |  |  |  |  |
| 2 | 0 | 0.99248 | - |  |  |  |  |  |  |  |  |
| 3 | 0.10525 | 0.0001 | 0.10079 | - |  |  |  |  |  |  |  |
| 4 | 0.95624 | 0.66069 | 0.95089 | 0.91277 | - |  |  |  |  |  |  |
| 5 | 0.62406 | 0.06931 | 0.62376 | 0.02406 | 1 | - |  |  |  |  |  |
| 6 | 0.90782 | 0.12683 | 0.90584 | 0.01 | 0.85248 | 0.15129 | - |  |  |  |  |
| 7 | 0.09584 | 0.14396 | 0.13238 | 0.00406 | 1 | 0.12733 | 0.00842 | - |  |  |  |
| 8 | 0.72218 | 0.54228 | 0.72436 | 0.69079 | 0.17158 | 0.90545 | 0.46802 | 0.1499 | - |  |  |
| 9 | 0.86119 | 0.99376 | 0.86525 | 0.54644 | 0.14059 | 1 | 0.65762 | 0.47574 | 0.03733 | - |  |
| 10 | 0.60574 | 0.83782 | 0.6096 | 0.69218 | 0.05426 | 0.17317 | 0.17881 | 1 | 0.0195 | 0.00277 | - |
